# Inequalities in the psychological well-being of employed, single and partnered mothers: the role of psychosocial work quality and work-family conflict

**DOI:** 10.1186/1475-9276-9-6

**Published:** 2010-02-22

**Authors:** Ewelina Dziak, Bonnie L Janzen, Nazeem Muhajarine

**Affiliations:** 1 Department of Community Health & Epidemiology, University of Saskatchewan, Health Sciences Building, 107 Wiggins Road, Saskatoon, Saskatchewan, S7N 5E5, Canada; 2 Saskatchewan Population Health and Evaluation Research Unit, University of Saskatchewan, Canada

## Abstract

**Background:**

A large body of international research reveals that single mothers experience poorer mental health than their partnered counterparts, with socioeconomic disadvantage identified as an important contributory factor in understanding this health disparity. Much less research, however, has focused specifically on the psychological well-being of single mothers who are employed, despite their growing presence in the labor force. Of the research which has considered employment, the focus has been on employment status *per se *rather than on other important work-related factors which may impact psychological health, such as psychosocial work quality and work-family conflict. The aim of this study was to: (1) compare employed single mothers and employed partnered mothers on measures of psychological distress, psychosocial work quality and work-family conflict; and (2) explore the potential role of work-family conflict and psychosocial work quality as explanations for any observed differences in psychological distress based on partner status.

**Method:**

Analysis of data obtained from a cross-sectional telephone survey of employed parents in a mid-sized Western Canadian city. Analyses were based on 674 employed mothers (438 partnered and 236 single), who were 25-50 years old, with at least one child in the household.

**Results:**

Compared to employed single mothers, employed partnered mothers were older, had more education and reported fewer hours of paid work. Single mothers reported higher levels of psychological distress, financial hardship, work-family conflict and poor psychosocial work quality. Statistical adjustment for income adequacy, psychosocial work quality and work-family conflict each independently resulted in single motherhood no longer being associated with psychological distress.

**Conclusions:**

While single employed mothers did experience higher levels of psychological distress than their partnered counterparts, differences between these groups of women in income adequacy, psychosocial work quality, and work-family conflict were found to explain this relationship. Future research employing a longitudinal design and subject to lower selection biases is required to tease out the interrelationship of these three life strains and to point to the most appropriate economic and social policies to support single mothers in the workforce.

## Background

Decreasing marriage rates and increasing divorce rates in North America over the last three decades have resulted in substantial growth in the number of single parent families [1]. In Canada in 2006, single parent families accounted for 16% of all families, up from 11% in 1981 [2]. Single parent families are overwhelmingly led by women, comprising approximately 80% of all such Canadian families in 2006. A large body of international research reveals that single mothers experience poorer mental and physical health than their partnered counterparts [3-6]. This health differential has been largely attributed to the chronic economic and social stressors to which many single mothers are exposed [7-9]. Less research, however, has focused on the well-being of single mothers who are employed, despite their growing presence in the Canadian labour force [1]. In 2004, just over two thirds of Canadian single mothers were employed, compared with less than half in 1976.

Of the research which has considered employment in relation to the health of single mothers, the focus has been on employment status *per se *[10] rather than on other important employment-related characteristics which may impact well-being, such as work quality. A number of conceptual models have been developed which highlight the importance of the psychosocial work environment in the health of employed adults, among the most popular being Robert Karasek's Job Strain Model [11,12]. Within this framework, workers' psychological job demands (e.g., pace, effort, volume) interact with their level of decision latitude (e.g. ability to make decisions at work and opportunity to use skills) to determine the psychosocial quality of their work. Job strain occurs when the psychological demands of the job are high and the worker's decision latitude (i.e., job control) is low. A considerable body of research has linked job strain, low job control, and high psychological demands with an increased risk of a variety of physical and mental health problems [12,13]. The fact that single mothers are, on average, more likely than partnered mothers to be employed in lower status, lower paying jobs [14] may also increase single mothers' exposure to poor psychosocial working conditions, and hence, their risk of psychological distress. To our knowledge, no research to date has systematically compared the psychosocial paid work environment of single and partnered mothers using Karasek's job strain typology, nor to what extent such differences may assist in explaining family structure disparities in mental health.

Employed single mothers may also experience greater challenges than partnered women in simultaneously negotiating family responsibilities and workplace demands [15,16]. Work-family conflict is defined as "*a type of inter-role conflict that occurs as a result of incompatible role pressures from the work and family domains*" [[17]; p.77]. Current frameworks underscore the bidirectional nature of work-family conflict in that family demands can conflict with work responsibilities, that is, family-to-work conflict, and work demands can conflict with family responsibilities, that is, work-to-family conflict [18,19]. In addition, different types of work-family conflict have been identified, the two most common being time-based and strain-based conflict. Conflict between work and family life, particularly strain-based, work-to-family conflict, has been associated with a number of negative physical and mental health outcomes [18,20]. However, this body of research has predominantly drawn on the experiences of those in dual-earner households, while studies involving the efforts of single mothers to negotiate work-family balance, and the potential impact of that struggle on their well-being, have been sparse [21]. Although a very limited amount of research suggests that employed single mothers may experience higher levels of work-family conflict than partnered mothers, these studies suffer from important methodological limitations, most notably the use of single item measures of work family conflict [16] or study specific scales with unknown psychometric properties which fail to adequately capture the different forms of work-family conflict [22]. A growing body of research suggests that the antecedents of work-family conflict differ for the different types of work-family conflict [23,24]. It is possible that single and partnered mothers may differ on some but not all aspects of work-family conflict, which would have implications for informing specific policies designed to enhance work-life balance.

The aim of this study, then, was to compare levels of psychological distress in employed single mothers relative to partnered mothers and to explore the potential role of work-family conflict and psychosocial job quality as explanations for any observed differences in psychological distress.

## Methods

### Sample

Data for the present study is based on a work, family and health telephone survey conducted in a mid-size Canadian city during 2005. Sample eligibility was limited to those who were: 1) English-speaking, 2) between the ages of 25 and 50 years, 3) employed, and 4) the parent of at least one child under the age of 20 years living in the household. In addition to these primary selection criteria, our goal was to sample a heterogeneous cross-section of employed parents according to age, job characteristics and economic circumstances. Toward this end, a data collection grid was developed to ensure that approximately equal numbers of participants were included in the final sample in terms of gender, age group (25-34 yrs; 35-50 yrs), and education (high school or less; some postsecondary; university/college degree). Trained interviewers randomly dialed the phone numbers; in households with more than one eligible person, one person was randomly selected to be interviewed. Telephone interviews averaged 40 minutes in length and were conducted using a computer-assisted telephone interviewing system. The study was approved by the University's Behavioural Research Ethics Board.

Of the 5300 eligible individuals contacted, 1160 were interviewed successfully, giving a response rate of 22%. Our sample was 58% female and one-half of the respondents were under the age of 35 years. The number of respondents was evenly distributed across the three educational groupings, with one-third reporting an educational attainment of high school or less. To examine the potential for selection bias, we compared the distribution of our respondents' answers with those from the Canadian Community Health Survey, Cycle 3.1 (CCHS 3.1) on similar questions [25]. Our comparison was restricted to CCHS respondents who were residing in the same city as our participants, employed, of similar age, and who were the parent of at least one child living in the household. Compared to respondents from the CCHS 3.1, our sample was younger and had lower educational attainment. Although our respondents reported more psychological distress, no statistically significant differences emerged between our sample and the national survey sample in terms of gender, work hours, self-rated health, or in the proportion reporting at least one chronic health condition.

To meet the research objectives of the present study, analyses were restricted to 674 employed mothers (438 partnered and 236 single).

### Variables

The dependent variable, psychological distress, was measured by the Kessler-6 (K6), a 6-item self-report measure requiring respondents to estimate on a 5-point response scale (0 = none of the time to 4 = all of the time) how often in the past 30 days they had experienced various symptoms of psychological distress [26]. Items include "How often in the past 30 days did you feel so depressed that nothing could cheer you up?", "How often did you feel hopeless?", and "How often did you feel restless or fidgety?" Respondents' scores were totaled across all the items, with higher scores indicating higher levels of psychological distress. In the present study, Cronbach's alpha for the scale was .80. The K6 has been shown to be a sensitive screen for DSM-IV disorders in general population samples [27,28].

The primary independent variable in this study, partner status, was a dichotomous variable based on current marital status. Partnered women were those who indicated that they were married or living with a partner. Unpartnered women were those who were separated, divorced, widowed, or never married. The three categories of unpartnered status were collapsed into a single group based on analyses indicating the absence of any statistically significant differences between groups on psychological distress, psychosocial job quality, or work-family conflict.

Psychosocial job quality was measured using Karasek's Job Content Questionnaire [11,12]. Items on the questionnaire combine to form several subscales reflecting key aspects of job quality. Decision latitude (9 items) consists of two dimensions: 1) decision authority (i.e., authority to make decisions concerning work); and 2) skill discretion (i.e., ability to use one's skills in doing work). Psychological job demands (10 items) refer to "how hard workers work" (e.g., pace, effort, and volume of work) and the presence of conflicting demands. The questionnaire items were coded from 1 *(*strongly agree) to 4 (strongly disagree) according to the degree to which respondents agreed with each statement. All items were recoded in the same direction, and scores for each scale were calculated by summing the item scores. A higher score for each scale indicates greater job demands and decision latitude. Cronbach's alpha for decision latitude and job demands was .74 and .64, respectively. To better represent Karasek's proposed model of job strain, participants' scores on the job demands and decision latitude scales were then categorized using median splits [29,30], resulting in four dimensions of psychosocial work quality: high strain (high job demands/low decision latitude), low strain (low job demands, high decision latitude), active (high job demands, high decision latitude) and passive (low job demands, low decision latitude). Evidence in support of the validity and reliability of the JCQ scales has been reported in numerous international studies [12].

Work-family conflict was assessed by a 12-item scale in which respondents were asked to indicate their agreement (1 = strongly disagree to 5 = strongly agree) with various statements related to work-family conflict [19]. Responses were summed to form four subscales (with three items each) measuring different types and directions of work-family conflict: 1) time-based work-to-family conflict (e.g., "My work keeps me from my family activities more than I would like"); 2) time-based family-to-work conflict (e.g., "The time I spend on family responsibilities often interfere with my work responsibilities"); 3) strain-based work-to-family conflict (e.g., "When I get home from work I am often too frazzled to participate in family activities"), and 4) strain-based family-to-work conflict (e.g., "Tension and anxiety from my family life often weakens my ability to do my job"). Higher scores reflect greater perceived work-family conflict. Cronbach's alpha for each subscale ranged between .84 and .86. Evidence of the scales' discriminant validity and internal consistency has been reported in previous research [19,31].

Several economic and demographic variables were also considered. Measures of socioeconomic position included educational attainment (high school graduate or less, some post-secondary training, or college/university graduate) and perceived income adequacy. Perceived income adequacy was assessed with one statement ("We have enough money to cover basic needs for food, housing and clothing") with which participants were asked to indicate their agreement on a scale from one (strongly disagree) to five (strongly agree). Higher scores indicated greater perceived income adequacy. Demographic characteristics included mothers' age, number of children, weekly work hours and the presence of at least one child age five years or younger in the household.

### Statistical Analyses

Data analyses involved a multi-stage process consisting of univariate, bivariate, and multivariable analyses using SPSS 15.0. Bivariate analyses were conducted to examine the demographic, social, and mental health characteristics of the study participants according to partner status. Differences between single and partnered mothers were tested using chi-square tests for categorical variables and t-tests for continuous measures.

Two sets of multiple linear regression analyses were conducted to examine whether any observed difference in psychological distress between single and partnered mothers could be accounted for by our study variables. In the first set of analyses, Model 1 assessed the unadjusted association between partner status and distress, with subsequent models evaluating the separate effect of each explanatory block of variables on this primary relationship of interest: Model 2, partner status plus demographic characteristics; Model 3, partner status plus socioeconomic factors; Model 4, partner status plus psychosocial work quality (high strain, low strain, active and passive); and Model 5, work-family conflict (time-based work-to-family conflict, time-based family-to-work conflict, strain-based work-to-family conflict, and strain-based family-to-work conflict). In Model 6, all variables were entered simultaneously into the regression equation. The second series of regressions followed the same process as the first, but rather than variable blocks, evaluated the separate effects of individual variables on the partner status - psychological distress association.

Preliminary analysis had indicated that the psychological distress variable and three of the work-family conflict variables (i.e., time-based family-to-work conflict, strain-based family-to-work conflict, and time-based work-to-family conflict) were positively skewed. These four variables were then square root transformed and examination of their revised distributions revealed that the shape of their respective distributions improved considerably. To further investigate the impact of the data transformations, linear regressions were conducted using both the raw and transformed scores of these variables and the results compared. Transformation of the three work-family conflict variables did not meaningfully impact the magnitude of the standardized beta coefficients leading to the decision to retain the variables in their original form to enhance interpretability. For psychological distress, however, raw and transformed comparisons revealed more pronounced differences in the beta coefficients; therefore, the square root transformed version of psychological distress was used in all regression analyses to improve concordance with statistical assumptions.

## Results

Intercorrelations among the variables were generally low; the work-family conflict variables were moderately correlated (.40 to .59) although not at a level approaching collinearity (data not shown). The key explanatory variables, according to partner status, are shown in Table 1. Compared to single mothers, partnered mothers were older, had more education, reported fewer hours of paid work, and were more likely to perceive an adequate household income. Although there were no differences in job demands, single mothers reported significantly lower levels of decision latitude than partnered mothers and a significantly higher percentage of single mothers were categorized as being in the high strain quadrant (ie., high job demands/low decision latitude). Compared with partnered mothers, single mothers scored significantly higher on time-based work-to-family conflict, strain-based work-to-family conflict, and strain-based family-to-work conflict. Finally, single mothers reported significantly higher levels of psychological distress than partnered mothers.

**Table 1:** Sociodemographic characteristics, psychosocial work characteristics, and work-family conflict, by partner status.

	Partnered Mothers (n = 438)	Single Mothers (n = 236)
	**%**	
Educational attainment**		
High school or less	28.8	39.8
Some postsecondary	29.0	29.2
College/university	42.2	30.9
		
Child ≤ 5 years of age living in household		
No	47.0	50.8
Yes	53.0	49.2
		
Psychosocial work quality**		
Low strain (low job demands/high decision latitude)	27.6	15.4
Passive (low job demands/low decision latitude)	24.4	22.4
Active (high job demands/high decision latitude)	29.9	35.5
High strain (high job demands, low decision latitude)	18.1	26.8
		
	**Mean (SD)**	
Number of children	2.02 (0.94)	1.88 (0.86)
Age**	36.69 (7.06)	35.15 (7.27)
Weekly work hours*	36.74 (10.68)	38.61 (11.32)
Perceived income adequacy**	3.31 (0.88)	2.88 (1.01)
Psychosocial work quality		
Decision latitude**	27.07 (4.77)	25.69 (4.82)
Psychological demands	24.39 (4.19)	25.03 (4.57)
Work-family conflict		
Time-based work-to-family**	7.00 (3.08)	8.11 (3.67)
Strain-based work-to-family**	6.84 (2.84)	7.49 (2.88)
Time-based family-to-work	6.05 (2.62)	6.43 (3.04)
Strain-based family-to- work*	5.70 (2.51)	6.12 (2.50)
Psychological distress*	3.90 (3.78)	4.50 (3.91)

p ≤ 0.05*

p ≤ 0.01**

### Linear Regression Analyses

Results of the regression analysis, first estimating the unadjusted association between partner status and psychological distress, then adjusting for different combinations of explanatory factors, are shown in Table 2. Model 1 indicates that single mothers had significantly higher levels of psychological distress than partnered mothers. In Model 2, the addition of demographic characteristics resulted in only a slight decrease in the beta coefficient, with single parent status remaining statistically significantly associated with psychological distress. The introduction of socioeconomic factors in Model 3 resulted in the beta coefficient for partner status decreasing by 50% and the relationship between partner status and psychological distress no longer statistically significant. Similar results occur with the addition of psychosocial work quality in Model 4 and work-family conflict in Model 5, with both variable blocks independently resulting in a reduction in the beta coefficient by 25% and no statistical association between partner status and psychological distress. With all of the explanatory factors included simultaneously in Model 6, the relationship between partner status and psychological distress remains non-significant.

**Table 2:** Associations between partner status and psychological distress, adjusting for various combinations of explanatory factors^a^.

	Model 1	Model 2	Model 3	Model 4	Model 5	Model 6
Unpartnered (compared to partnered)	0.09*	0.08*	0.04	0.06	0.06	-0.01
Age		-0.04				-0.06
Number of children		-0.10*				-0.08
Child ≤ 5 years of age living in household (compared to no young child)		-0.00				-0.03
Weekly work hours		-0.03				-0.07
Education (compared to university/college graduate)						
Some post-secondary			0.11*			0.10*
High school or less			0.04			0.05
Perceived income adequacy			-0.19**			-0.13**
Psychosocial work quality (compared to low strain)						
Active				0.12*		0.09
Passive				0.07		0.01
High strain				0.22**		0.12*
Work-family conflict						
Time-based work-to-family					-0.02	-0.01
Strain-based work-to-family					0.06	0.03
Time-based family-to-work					-0.01	-0.01
Strain-based family-to- work					0.27**	0.26**

^a^Note: standardized regression coefficients (beta) are reported

p ≤ 0.05*

p ≤ 0.01**

Table 3 shows associations between partner status and psychological distress, adjusting for the explanatory factors individually. For educational attainment (Model 2) and time-based family-to-work conflict (Model 8), the beta coefficients for partner status decline slightly but remain statistically significant. The remaining explanatory variables all reduce the association between partner status and psychological distress to statistical non-significance, with the largest independent attenuation occurring with the addition of income adequacy in Model 3.

**Table 3:** Associations between partner status and psychological distress, adjusting for individual explanatory factors.

	Standardized beta coefficient (partner status)
Model 1: partner status	.086*
Model 2: partner status and educational attainment	.081*
Model 3: partner status and income adequacy	.044
Model 4: partner status and psychosocial work quality	.055
Model 5: partner status and time-based work-to-family conflict	.066
Model 6: partner status and strain-based work-to-family conflict	.067
Model 7: partner status and strain-based family-to-work conflict	.062
Model 8: partner status and time-based family-to-work conflict	.075*

*p ≤ 0.05

## Discussion

Similar to the results of previous research with general population samples of single mothers [3,4,6], employed single mothers in this study reported significantly higher levels of psychological distress compared to their partnered counterparts. More importantly, however, the greater distress observed among single mothers' could be completely explained by their greater exposure than partnered mothers to financial strain, psychosocial work stress, and work-family conflict.

Although employment for Canadian single mothers has increased dramatically in recent decades, lower wages combined with the absence of a second earner means that they are still much more likely than partnered women to live in a low income household [32-34]. In 2007, the overall prevalence of low income among employed lone mothers in Canada was 17%, compared with 2% among employed mothers with partners. For single mothers in low paying jobs, approximately one-quarter of all Canadian employed single mothers, the prevalence of a low household income increases to 56%, compared to 14% among low wage partnered mothers [33]. Low wage jobs, in addition to a limited income, also means less access to non-wage benefits that can offset the expenses of raising a family, such as supplementary health insurance and dental plans [35]. In this study, although all of the single mothers were employed *and *reported working more hours per week than partnered mothers, they were significantly more likely to perceive that their income did not adequately cover their food, shelter and clothing expenses. The strain of financial hardship, that is, the perceived gap between one's basic needs and the resources available to meet those needs, has been linked with a variety of negative mental and physical health outcomes [36]. Importantly, after adjusting for perceived income adequacy in this study, being a single mother was no longer statistically associated with higher psychological distress. Our findings are consistent with previous research which has also highlighted financial hardship as an important contributory factor in the elevated psychological distress of single compared to partnered mothers [7-9].

Jobs vary not only in economic returns but in psychosocial quality as well. Employed single mothers are, on average, more likely to be working in lower paying, low-skilled jobs than partnered mothers [34,37]. In turn, lower skilled jobs tend to be associated with poorer psychosocial job characteristics [12]. According to Karasek's job strain model, the least physically and mentally healthy work environments are those in which demands are high and workers' ability to respond to those demands are low [11,12]. Poor psychosocial work conditions may impact mental health negatively by eroding workers feelings of self-worth and sense of mastery [38]. In the present study, employed single mothers reported lower levels of workplace decision latitude and a greater proportion of single than partnered mothers were in the high strain quadrant of Karasek's job strain typology (i.e., high job demands and low decision latitude). In addition, controlling for single mothers' greater exposure to work stress in the regression analysis reduced the association between partner status and psychological distress to one that was no longer statistically significant. Thus, the economic, social, and psychological benefits often associated with employment for mothers [39], may by diminished for single mothers when in jobs that involve little creativity or freedom in decision making.

Lower-status jobs may also provide less support for employees to meet family demands [40]. Work-family conflict is a perception of inadequate energy and/or time to effectively fulfill work and family obligations [17]. Several qualitative studies have documented single mothers' difficulties in their attempts to negotiate work and family responsibilities, particularly when in jobs that lack flexible work hours and family-supportive managers [15,41]. In addition to job characteristics, the absence of a resident partner, and presumably the instrumental and emotional support which would accompany that role, may also make it more difficult for single than partnered mothers to balance work and family demands. In this study, employed single mothers reported higher levels of three out of the four different forms of work-family conflict assessed, both from those pressures originating in the work place (ie., time/strain-based, work-to-family conflict) and those originating in the home environment (ie., strain-based, family to work conflict). Further, each type of work-family conflict was able to independently account for the statistically significant association between being a single mother and excess psychological distress. Research with general population samples has identified access to instrumental and emotional support, in both work and non-work domains, as key resources in reducing work-family conflict [24]. Importantly, support with managing the daily demands of family and work need not come from a resident partner: the findings from a recent qualitative study in the United States suggested that many of the women coped well as employed single mothers, drawing extensively on the support of their community and extended family ties for assistance [42].

### Study Strengths and Limitations

Although researchers have speculated that single parent status is likely associated with poorer psychosocial work quality and greater work-family conflict, our study is among the few to systematically examine these hypotheses and link such circumstances with disparities in psychological well-being. Another important strength is our use of theoretically-based and psychometrically-sound measures of psychosocial work quality and work-family conflict.

There were several limitations. All variables were based on self-reported measures, thus reporting biases cannot be ruled out. Also, access to potentially important information, such as custody arrangements and the duration of single parenthood, were not available in the present study. Among single mothers there is variability in terms of the other parent's level of involvement in their children's lives which would likely impact on the women's experience of single parenting. Also not addressed in the present study was the presence of other supportive people, such as grandparents, who may be available to assist working mothers in the daily demands of raising a family as a single parent.

In addition to the cross-sectional design, which impedes our understanding of the temporal relationship of study variables, the potential for response bias is an important concern. The lower than desired response rate is perhaps not completely surprising given that our target population - employed women with children - may have found it difficult to find the time in their busy schedules to actually participate in a study. Some evidence against bias is suggested by the fact that our analyses reproduced some of the factors that have already been "established" as being associated with psychological distress in previous research, including financial hardship, a high-strain work environment, and work-family conflict [18,29,43]. On the other hand, we cannot rule out the possibility that women who agreed to participate may have differed from nonparticipants in their perceptions of work, family and well-being. The positively skewed distribution of several of the work-family conflict variables may indicate that mothers who experienced higher levels of work-family conflict did not participate perhaps because of time constraints. In addition, although comparison with national Canadian survey data suggested that mothers in our study experienced, on average, more psychological distress, the positive skew of the data suggested that many of the women in our sample still experienced very low levels of psychological distress.

## Conclusions

While single employed mothers did experience higher levels of psychological distress than their partnered counterparts, differences between these groups of women in perceived income adequacy, psychosocial work quality, and work-family conflict were found to explain this relationship. Future research employing a longitudinal design and subject to lower selection biases is required to tease out the interrelationship of these three life strains and to point to the most appropriate economic, work and family policies to support employed single mothers.

## Competing interests

The authors declare that they have no competing interests.

## Authors' contributions

This manuscript is largely based on ED's master's thesis, which was conducted under the supervision of BLJ. BLJ designed the study, oversaw the statistical analyses, and prepared the manuscript. NM participated in the design of the study and assisted in drafting the manuscript. All authors read and approved the final manuscript.
